# Independence of Size and Distance in Binocular Vision

**DOI:** 10.3389/fpsyg.2018.00988

**Published:** 2018-06-25

**Authors:** Nam-Gyoon Kim

**Affiliations:** Department of Psychology, Keimyung University, Daegu, South Korea

**Keywords:** size perception, distance perception, size distance invariance hypothesis, binocular vision, interpupillary distance

## Abstract

For too long, the size distance invariance hypothesis (SDIH) has been the prevalent explanation for size perception. Despite inconclusive evidence, the SDIH has endured, primarily due to lack of suitable information sources for size perception. Because it was derived using the geometry of monocular viewing, another issue is whether the SDIH can encompass binocular vision. A possible alternative to SDIH now exists. The binocular source of size information proposed by Kim (2017) provides metric information about an object’s size. Comprised of four angular measures and the interpupillary distance (IPD), with the explicit exclusion of egocentric distance information, Kim’s binocular variable demands independence of perceived size and perceived distance, whereas the SDIH assumes interdependence of the two percepts. The validity of Kim’s proposed information source was tested in three experiments in which participants viewed a virtual object stereoscopically then judged its size and distance. In Experiments 1 and 2, participants’ size judgments were more accurate and less biased than their distance judgments, a finding further reinforced by the results of partial correlation analyses, demonstrating that perceived (stereoscopic) size and distance are independent, rather than interdependent as the SDIH assumes. Experiment 3 manipulated participants’ IPDs, one component of Kim’s proposed variable. Size and distance judgments were overestimated under a diminished IPD, but underestimated under an enlarged IPD, a result consistent with predictions based on participants’ utilization of the proposed information source. Results provide unequivocal evidence against the SDIH as an account of size perception and corroborate the utility of Kim’s proposed variable as a viable alternative for the binocular visual system.

## Introduction

The sense of solidity experienced when viewing a pair of two-dimensional (2-D) stereo images is compelling. The added depth that is unavailable in each 2-D image may contribute to the vivid impression. This may be why Pinker (1997, p. 241) declares binocular vision as “one of the glories of nature.” Indeed, it is well documented that binocular vision facilitates our daily interactions with the surrounding environment (Jackson et al., 1997; Watt and Bradshaw, 2000, 2003; Melmoth and Grant, 2006).

The advantages of binocular vision have been well recognized, but its contribution to space perception has been unimpressive. Of the many sources of spatial information identified to date, only two distance cues (convergence and binocular disparity) are binocular. Further, the efficacy of these two sources is rather limited, effective, at best, up to 2 m but no more than 6 m from the observer (Ono and Comerford, 1977, for review; but see Allison et al., 2009; Palmisano et al., 2010). Nevertheless, these two information sources carry extra significance. Convergence (i.e., inward or outward turning of the eyes to fixate objects at different distances) may well be the only cue that provides absolute metric information (Kaufman, 1974); whereas binocular disparity (i.e., the difference in the images of the two eyes due to their different viewpoints) provides the sense of solidity (i.e., three-dimensionality) of objects. Presumably, the limited ranges of the two binocular sources of distance information are supplemented by other monocular sources of distance information to yield accurate awareness of the surrounding layout in depth.

Distance perception abounds with various sources of information; but its counterpart, size perception, does not. In fact, to date only a few sources of information have been identified to account for size perception (e.g., familiar size, relative size, and horizon ratio). The disparity between the number of candidate information sources for size and distance is puzzling, given the long history of this problem (Ross and Plug, 1998; Hatfield, 2002). Even for those few sources of information that have been postulated to support accurate size perception, their efficacy is limited. For example, Haber and Levin (2001) demonstrated that familiar size can be an effective cue for size judgments. Interestingly, their participants were able to judge the sizes of unfamiliar objects with comparable precision. Unable to provide an adequate explanation for this finding, they lamented that “All we can say is that they did not do it in the same way as they did for the distance estimations. This ignorance reflects a general ignorance about the perceptual variables underlying size perception” (p. 1150).

The horizon ratio, first introduced by Sedgwick (1980), utilizes the fact that one’s eye height coincides with the horizon line. The absolute height of an object, therefore, can be determined in proportion to one’s eye height. Wraga (1999; see also Dixon et al., 2000) confirmed the utility of this information source by explicitly manipulating one’s perceived eye height by surreptitiously varying the floor height. Results showed that the perceived heights of objects varied in accordance with perceived eye height, positive evidence for the utility of the horizon ratio. Interestingly, eye height’s impact on judging object width was minimal. Based on these findings, Wraga concluded that eye height can be utilized as a natural metric for object height, but not for object width. Thus, it is safe to conclude that there has yet to be a comprehensive account of perceptual capacity for size judgments, in particular, the horizontal extents of objects, apart from the size distance invariance hypothesis (SDIH).

**FIGURE 1:**
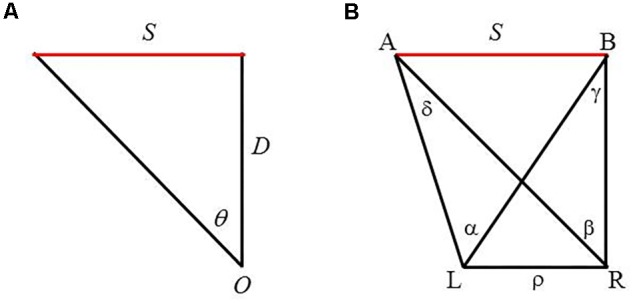
**(A)** Monocular geometry depicting the size distance invariance hypothesis (SDIH). An object of size *S* is at a distance *D* from an observer *O*, thus subtends a visual angle 𝜃. **(B)** Binocular geometry for viewing a line segment *AB*. L and R refer to the left and right eye, respectively, and ρ the IPD. A and B are the two end points of the line segment. α and β are visual angles subtended by *AB* with respect to each eye, whereas γ and δ are binocular parallaxes of each end point of the segment with respect to the two eyes.

Indeed, the prevalent explanation for size perception has been the SDIH. As illustrated in **Figure 1A**, the two sides of the triangle, *S* and *D*, are inversely related to the angle, 𝜃, through a trigonometric relation, tan 𝜃 = *S*/*D*. Extending this geometric relation to perception, the hypothesis states that the visual angle 𝜃 subtended by an object determines a unique ratio of the perceived size of the object *S*′ to its perceived distance *D*′, that is, tan 𝜃 = *S*′/*D*′ (Kilpatrick and Ittelson, 1953; Epstein et al., 1961).

An infinite number of size and distance combinations exists for any given angle. Yet, it is primarily perceived size, not perceived distance, for which the SDIH is utilized. Lack of identified information sources for size perception may have contributed to this biased application of the SDIH. Hence, the perceived size of an object is thought to be determined by both the visual angle the object subtends and its perceived distance, that is, *S*′ = *D*′ tan 𝜃. The conjecture that perceived size is derived from visual angle by taking perceived distance into account has been referred to as the “taking-into-account” model (Epstein, 1973, 1977; Higashiyama and Shimono, 2004; Higashiyama and Adachi, 2006).

For the last several decades, extensive efforts have been made to validate the SDIH empirically. The results have been largely inconclusive, primarily due to anomalous effects collectively known as the *size-distance paradox* (see Ross, 2003, for review). Gruber (1954) set out to determine whether perceived size is proportional to perceived distance when image-size is held constant. He observed, instead, that “an object which is consistently underestimated in relative size was consistently overestimated in relative distance” (p. 426), a pattern opposite to that predicted by the SDIH. This effect has been replicated repeatedly (Heinemann et al., 1959; Baird and Biersdorf, 1967; Epstein and Landauer, 1969; Ono et al., 1974; Foley, 1980; Collewijn and Erkelens, 1990; Brenner and van Damme, 1998; see Ross, 2003, for a review).

Apart from the issue of being an effective account of size perception, the SDIH raises another issue, that is, whether it can be utilized as an account of size perception for binocular vision. As depicted in **Figure 1A**, the SDIH is derived based on the geometry of monocular viewing. However, there is ample evidence of the benefits of binocular vision in our daily interactions with the surrounding environment (Jackson et al., 1997; Watt and Bradshaw, 2000, 2003; Melmoth and Grant, 2006). As an illustration, to reach and grasp an object in space, the hand must be transported to the object of interest while the grip aperture must match the dimensions of the object. The transport component relies on extrinsic properties of the object (e.g., object’s distance); but the grasp component relies on intrinsic properties (e.g., size and shape). Watt and Bradshaw (2000) reported that removal of binocular information affected the formation of the grip aperture, but had negligible impact on the transport component.

Despite convincing demonstration that binocular vision facilitates the control of grasp, these researchers failed to identify the source of binocular information that facilitated the size judgments needed for the control of grasp. Thus, the question can be raised as to the exact source of binocular information that their participants utilized to control their grasp. Did their participants perceive an object’s distance first, then use that distance to recalibrate the retinal image to determine object size, in accordance with the SDIH; or did they utilize an as yet unknown binocular information source to judge object size?

Recently, Kim (2017) proposed an alternative source of information that the binocular visual system could utilize to detect an object’s size—the horizontal extent of an object. Drawing on the binocular geometry of viewing a fronto-parallel line segment AB (**Figure 1B**), the proposed variable is expressed as follows:

AB = ρsin αsin δsin βsin γ

Provided that the visual system can access its interpupillary distance (IPD), the contention is that any frontal size can, in principle, be perceived binocularly based on the proposed binocular variable. As is the case with convergence angle, the IPD provides a metric basis, enabling the variable to convey absolute metric information about object size. More importantly, the information for an object’s size, according to this binocular variable, is directly available in optical stimulation, even in the absence of egocentric distance information. Hence, its utility necessarily demands the independence of the perceptions of size and distance.

As da Vinci noted five centuries ago, the binocular mode of visual perception may be fundamentally different from the monocular mode of visual perception, particularly at short distances (see Wade et al., 2001, for further details; see also Ono et al., 2002). Yet, research on binocular vision has relied exclusively on a description developed based on the viewing geometry of monocular vision, in particular, the SDIH. This is problematic. The present study set out to determine whether the binocular visual system utilizes the SDIH or an alternative source information, such as that proposed by Kim (2017), to perceive an object’s size. To this end, three experiments were conducted in which participants viewed a virtual object stereoscopically then judged its size and distance. The first two experiments ascertained whether perceived (stereoscopic) size and perceived (stereoscopic) distance are interdependent, as predicted by the SDIH, or independent, as entailed by Kim’s (2017) proposed binocular information source. The results showed little evidence that the two perceptual qualities are related, thus contradicting the SDIH. Because these results can only be construed as indirect evidence for the utility of the proposed information source, the third experiment sought direct evidence for its utility by manipulating the IPD, one component of the proposed variable.

## Experiment 1: Perceptual Independence of Size and Distance in Stereoscopic Vision

In research on size perception, the doctrine of size distance invariance has rarely been challenged. Any deviant results have been attributed to the degraded qualities of distance information. If, however, the perceptions of size and of distance are independent, as hypothesized here, a different method will be needed to evaluate this hypothesis. Garner and Morton (1969; see also Ashby and Townsend, 1986; Amazeen, 1999) caution that a proper experimental paradigm to test the perceptual independence of two percepts must be one in which the two stimulus variables are controlled independently and the corresponding perceptions are assessed separately. Only then can a lack of independence in performance be attributed to limitations in the perceiver rather than to limitations in the experimental arrangement.

In the current experiment, participants watched virtual images of a cube of varying size under stereoscopic viewing conditions. The images were rendered in cross disparity so that they appeared to be floating in front of the computer monitor at varying distances from the observer. Participants reported the perceived location of the image, as well as its horizontal extent, by manipulating the reporting apparatus with their right hands, which were hidden from their view (see below for more details).

### Participants

Nineteen undergraduates (1 male and 18 female) from Keimyung University volunteered for the experiment and received course credit for their participation. All participants had normal or corrected-to-normal vision. With the exception of one participant, all had normal stereoacuity of at least 100 s of arc, as measured by the Multi-Target Red/Green Anaglyph Stereo Test (Random Dot Butterfly, Letter “E,” and Figures; Synthetic Optics Inc., Franklin Lakes, NJ, United States). Her data were excluded from analysis.

### Ethics Statement

The study was approved by the Keimyung University’s Institutional Review Board. After providing a complete description of the study to the participants, written informed consent was obtained in accordance with the Declaration of Helsinki.

### Apparatus

The visual stimuli were generated on a Dell Precision 380 workstation equipped with an NVIDIA Quadro FX3450 graphics card (NVIDIA, Santa Clara, CA, United States) and presented on a 21-in Samsung SyncMaster Magic CD210JP CRT monitor refreshed at 120 Hz. Participants viewed the displays in a dimly lit room through CrystalEyes (StereoGraphics, San Rafael, CA, United States) liquid crystal (LC) shutter glasses that were synchronized with the monitor’s refresh rate, which alternated at 60 Hz. The display had a resolution of 1080 H × 768 V pixels and subtended a field of view of 37.0° H × 28.0° V when viewed from a distance of 60 cm. A head and chin rest was used to restrict head movements.

**FIGURE 2:**
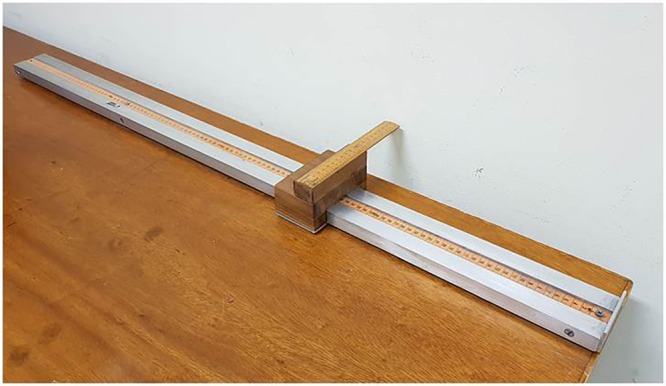
The reporting apparatus employed in the present study.

A 56-cm H × 75-cm V × 100-cm deep (D) matte black viewing box was placed between participants and the monitor. The viewing box had a window for the monitor on one end and a window for a chin rest at the other. In addition to enhancing stereoscopic viewing, the viewing box blocked participants’ views of the hand with which they reported their perceptions of distance and size for a virtual object. To record their responses, participants used a special reporting apparatus (**Figure 2**). A wooden track onto which a 120-cm ruler was placed was positioned parallel to participants’ lines of sight and to the right of the viewing box. A wooden block could be moved along the track. On the block there was another 20.5-cm ruler lying parallel to the observer’s frontal plane. The ruler on the track was used to report the perceived distance of the virtual object, whereas the short ruler on the block was used to report the perceived size of the object (see below for details).

### Stimuli

**FIGURE 3:**
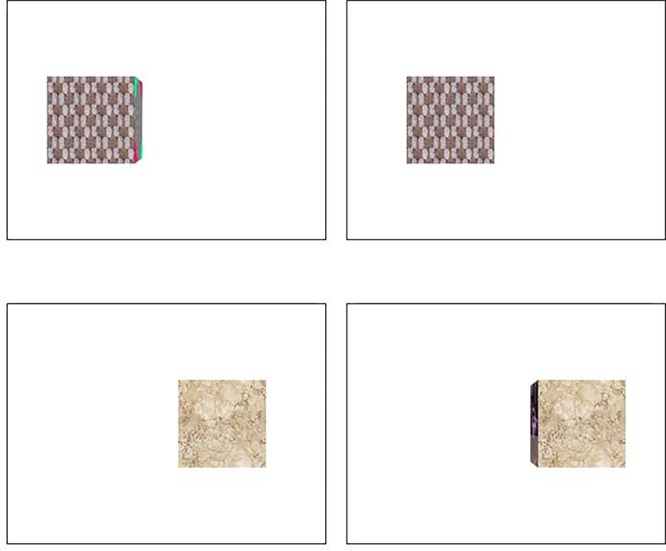
Stereograms used in the volumetric condition. All the stereo pairs rendered in cross disparity. Hence, the left pair is the image for the right eye, whereas the right pair is the image for the left eye.

The stimulus was a cube in which each of its six sides was rendered with a different texture. The cube was displayed against a white background (**Figure 3**). The six texture images were randomized in each trial to produce different images of the cube across trials to eliminate effects of familiarity and texture information on size judgments. The stereo images were calibrated in accordance with each participant’s IPD.

The stimulus object appeared either in its entirety as a three-dimensional (3-D) cube (the volumetric condition) or presenting only the frontal face as a 2-D rectangle (the frontal face condition). As part of control, each object appeared slightly to the left or to the right from the center of the screen. An additional effect of this manipulation was that each object, particularly in the volumetric condition, was depicted such that only its frontal face was projected to one eye, whereas the frontal face and one of the side panels were projected to the opposing eye, depending on the geometry of viewing (**Figure 3**). Note that in the volumetric condition, the object was rendered under perspective projection. Thus, despite the drastic difference in the two stereo images in the volumetric condition, when fused properly, the three-dimensionality of the perceived object in conjunction with its side panel rendered in perspective will further enhance depth impression.^1^ If the SDIH is utilized for size perception, performance should be facilitated more so by enhanced depth impression under the volumetric condition than under the frontal face condition.^2^

### Design

Four variables were manipulated. The simulated size of the cube varied among 3, 5, 7, 9, and 11 cm; and its simulated location varied among 34, 37, 40, 43, and 46 cm from the observation point. In the volumetric condition, the entire cube was shown; in the frontal face condition, only the frontal face of the cube was visible. Each cube was centered 3–4 cm to the left or to the right of the center of the screen. These manipulations yielded a 5 (Size) × 5 (Distance) × 2 (Shape) × 2 (Side) repeated measures design with 100 completely randomized trials.

### Procedure

The experiment employed a double-blind procedure in which the laboratory assistant who ran the experiment was naïve as to the purpose of the experiment, as were the participants. Upon presentation of each stereo image pair, participants were instructed to move the wooden block along the track and place the surface of the block facing them coincident with the front face of the cube or the front face of the flat surface, depending on the condition. Participants were encouraged to adjust the block until satisfied with their judgments. They were then instructed to indicate the frontal size of the object using their thumb and the index finger, with the tip of their thumbnails placed at the left end of the short ruler on the block. The tick mark on the ruler indicated by the tip of the index fingernail was used to determine the size of the object. No feedback was provided. While reporting their responses, participants were instructed to keep their heads inside the viewing box and maintain their gaze on the monitor. Thus, the scales read by the experimenter were not visible to participants.

### Data Analysis

First, performance was assessed in terms of constant error^3^ employing an analysis of variance (ANOVA) on each perceived quality, i.e., *S*′ (perceived size) and *D*′ (perceived distance) with size, distance, shape, and side as independent variables. Then, following Oyama (1974, 1977; see also Higashiyama and Shimono, 2004; Higashiyama and Adachi, 2006), partial correlation analyses were performed to assess the relationships among manipulated variables and perceptual variables. Partial correlation measures the relationship between two variables while holding the effect of other variables constant. Oyama contends that the patterns of partial correlations can be used to infer causal relations among variables. For example, given three variables, *X, Y*, and *Z* with the assumption that *X* is always an independent variable and every relation between two variables is linear, if *X* determines *Y* and *Y* determines *Z*, that is, *Y* is mediating *X* to determine *Z*, a high bivariate correlation between *X* and *Z* becomes almost zero when the effect of *Y* is removed. If, as the SDIH predicts, *S*′ is derived from visual angle 𝜃 by taking *D*′ into account, the partial correlation between 𝜃 and *S*′ should be zero when the effect of *D*′ is controlled. If, on the other hand, as contended here, *S*′ and *D*′ are independent, or more specifically, *S*′ is directly perceived by the information conveyed by Kim’s (2017) binocular variable, a high bivariate correlation between *S*′ and object size *S* should remain unaffected even when the influences of *D*′ or any other candidate intervening variables, such as 𝜃 or convergence angle *ϕ*, are held constant. For correlation analyses, *𝜃*, ϕ, *S*, and *D* were entered as stimulus (or manipulated) variables^4^ and *S*′ and *D*′ as perceptual (or responding) variables. *S* is assumed to be specified by the proposed information source and *D* either by *ϕ* or a yet to be discovered higher-order optical variable specifying distance information binocularly comparable to Kim’s (2017) binocular size variable. Except for binocular disparity and convergence angle, all other spatial cues were unavailable under the experimental setup, that is, viewing stereoscopically produced virtual images of variously sized cubes rendered in novel texture images. Because these two distance cues are perfectly correlated with each other, only convergence angle was entered in the correlation analyses as distance information.

### Results and Discussion

The five object sizes employed in the experiment were 3, 5, 7, 9, and 11 cm; and the means of the corresponding perceived sizes (*SD*) were 5.23 (0.84), 7.43 (1.00), 9.38 (1.25), 11.43 (1.54), and 13.32 (1.66) cm, respectively. The five target locations employed were 34, 37, 40, 43, and 46 cm; and the means of the corresponding perceived distances (*SD*) were 23.37 (8.07), 25.76 (7.98), 27.53 (8.40), 29.85 (8.09), and 32.22 (7.79) cm, respectively.

Overall, participants performed poorly, overestimating size and underestimating distance. The overall mean constant errors in size and distance judgments were 2.36 cm (*SD* = 1.18) and -12.26 cm (*SD* = 7.93), respectively. Nevertheless, an ANOVA on perceived size with size as a within-subject factor confirmed a significant effect of size, *F*(4,68) = 502.88, *p* < 0.0001, $\eta_{p}^{2}$ = 0.97. A Tukey *post hoc* test confirmed that all five sizes were discriminated from one another at the 0.01 significance level. The result was the same for perceived distance, *F*(4,68) = 76.55, *p* < 0.0001, $\eta_{p}^{2}$ = 0.82; means at the tested distance values were significantly different from each other at the 0.01 level. It appears that, despite over- and underestimation of size and distance, participants responded systematically to the variables manipulated in the experiment.

#### Constant Error Analysis

**FIGURE 4:**
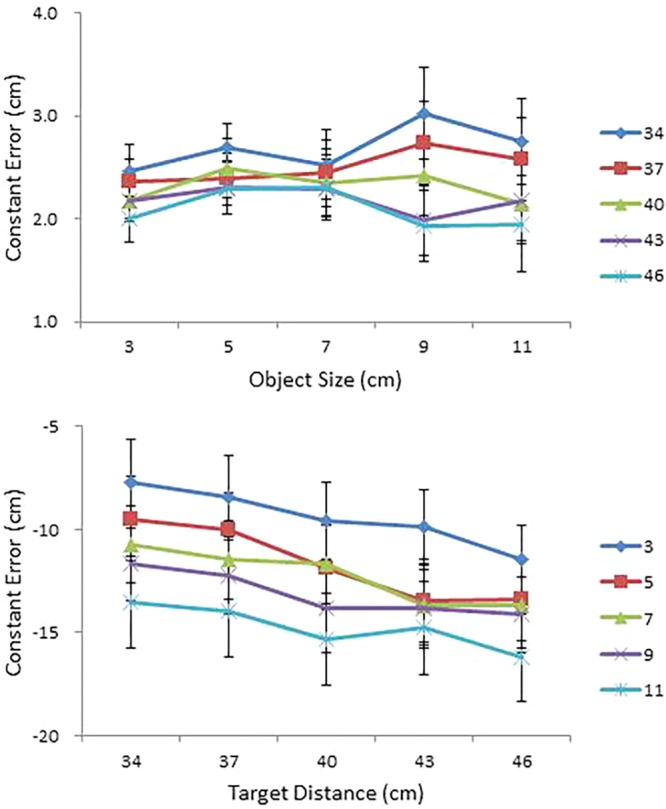
Mean constant error in perceived size as a function of object size (cm) for each condition of object distance (cm) **(top)**; and mean constant error in perceived distance as a function of object distance (cm) for each condition of object size (cm) **(bottom)** in Experiment 1. Error bars represent ±1 standard error (SE) of the mean.

Judgment accuracy was assessed using constant error. Mean constant error in perceived size and in perceived distance are presented, respectively, as a function of object size (cm) for each condition of object distance (cm) and as a function of object distance (cm) for each condition of object size (cm) in the top and bottom panels of **Figure 4**. An ANOVA on perceived size revealed a main effect of distance, *F*(4,68) = 15.58, *p* < 0.0001, $\eta_{p}^{2}$ = 0.48, a significant Size × Distance interaction, *F*(16,272) = 1.80, *p* < 0.05, $\eta_{p}^{2}$ = 0.10 (top panel of **Figure 4**), a significant Size × Shape interaction, *F*(4,68) = 3.68, *p* < 0.01, $\eta_{p}^{2}$ = 0.18, and a significant four-way interaction among size, distance, shape, and side, *F*(16,272) = 1.92, *p* < 0.05, $\eta_{p}^{2}$ = 0.10.

With respect to the main effect of distance, a Tukey *post hoc* test confirmed performance differences between the 34 cm condition and the 40, 43, and 46 cm conditions and between the 37 cm condition and the 43 and 46 cm conditions. Size overestimates tended to be greater at near distances (the 34 and 37 cm conditions) than at far distances (the 43 and 46 cm conditions). With respect to the Size × Distance interaction, a simple effects analysis confirmed that the effect of distance was significant in the 9 cm condition, *F*(4,14) = 6.31, *p* < 0.01, and in the 11 cm condition, *F*(4,14) = 3.85, *p* < 0.05. Taken together, overestimation of object size at near distances was particularly pronounced in the two largest (9 and 11 cm) size conditions (top panel of **Figure 4**). With respect to the Size × Shape interaction, a simple effects analysis revealed a significant effect of shape in the 9 cm size condition, *F*(1,17) = 6.00, *p* < 0.05.

The ANOVA on perceived distance confirmed main effects of size, *F*(4,68) = 17.03, *p* < 0.0001, $\eta_{p}^{2}$ = 0.50, distance, *F*(4,68) = 11.04, *p* < 0.0001, $\eta_{p}^{2}$ = 0.39, shape, *F*(1,17) = 5.94, *p* < 0.05, $\eta_{p}^{2}$ = 0.26, and side, *F*(1,17) = 5.18, *p* < 0.05, $\eta_{p}^{2}$ = 0.23. The ANOVA also confirmed a significant Distance × Shape interaction, *F*(4,68) = 5.42, *p* < 0.01, $\eta_{p}^{2}$ = 0.24, and a significant Distance × Size × Side interaction, *F*(16,272) = 2.12, *p* < 0.01, $\eta_{p}^{2}$ = 0.11.

All in all, distances were severely underestimated (that is, objects were perceived closer than they were), with the degree of underestimation magnified with increases in distance and in size. A Tukey test for the size effect confirmed performance differences between the 3 cm condition and the other four larger size conditions and between the 11 cm condition and the 5 and 7 cm conditions; whereas a Tukey test for the distance effect confirmed performance differences between the 34 cm condition and the 40, 43, and 46 cm conditions and between the 37 cm condition and the 43 and 46 cm conditions.

The degree of distance underestimation also increased when the virtual object was displayed slightly to the right of the center of the screen. Recall that participants reported their responses using the reporting apparatus that was located on their right-hand side. This asymmetry may have contributed to this effect, although it is not clear why objects appearing on the right side were more underestimated (i.e., judged closer) than those on the left.^5^

The same pattern of underestimation occurred for the shape of the object with more pronounced underestimation for 3-D objects (*M* = -0.56) than for 2-D objects (*M* = 0.44). Shape further interacted with distance. A simple effects analysis revealed that the effect of distance was significant in the frontal size condition, *F*(4,14) = 3.85, *p* < 0.05, and in the volumetric condition, *F*(4,14) = 7.21, *p* < 0.01; whereas the effect of shape was significant only at 40 cm, *F*(1,17) = 13.35, *p* < 0.01. It appears that perceived distances of virtual objects located 40 cm away from the observation point were underestimated less than those of objects located at other distances, especially when the objects appeared in 2-D (the frontal face condition) rather than in 3-D (the volumetric condition). The reason for this is unknown. Taken together, the effect of the shape of the virtual image on perception appears to be minimal on size judgments. Thus, the present result in which enhanced depth impression failed to facilitate size judgments contradicts, or at least is inconsistent with, the SDIH.

#### Correlational Analysis

Bivariate and partial correlation analyses were performed by pairing one of the four stimulus variables (i.e., 𝜃, ϕ, *S*, and *D*) with one of two perceptual variables (i.e., *S*′, *D*′) to explore causal relationships between these variables. The analyses were performed for each participant, and the mean coefficients are presented in **Table 1**. At first blush, strong correlations between 𝜃 and *S*′, *S* and *S*′, *D* and *D*′, and ϕ and *D*′ are easily noticeable. Interestingly, except for the *S* and *S*′ pair, other pairs’ correlations became zero when the effect of control variables was factored out. First, with respect to the 𝜃 and *S*′ pair, as Oyama (1974, 1977) suggests, the near zero partial correlation indicates a causal relationship between these two variables, a result corroborating the SDIH. Note that four variables (i.e., ϕ, *S, D*, and *D*′) were entered in the partial correlation computation. Thus, it is important to identify the exact source of the confounding effect to verify this possibility. A separate partial correlation was performed while controlling one variable at a time. The additional analysis revealed coefficients of 0.78 with *D*, 0.81 with *D*′, 0.81 with ϕ, and 0.09 with *S*, as the control variable, respectively. Clearly, it was *S* that confounded the relationship between 𝜃 and *S*′, not a distance related variable, i.e., ϕ, *D*, or *D*′. The apparent relation between 𝜃 and *S*′ was spurious, arising largely because both were highly correlated with *S*, not causal, as it would be if it were to corroborate the SDIH.

**Table 1:** Mean bivariate and partial correlation coefficients among stimulus and perceptual variables in Experiments 1 and 2.

		Experiment 1		Experiment 2	
Paired variables	Controlled variables	Bivariate correlation	Partial correlation	Bivariate correlation	Partial correlation
*S′, D′*	*S, D*, 𝜃, ϕ	–0.28	0.06	–0.23	0.09
*S′*, 𝜃	*S, D, D′*, ϕ	0.84	–0.02	0.83	0.02
*S′*, ϕ	*S, D, D′*, 𝜃	0.07	0.04	0.03	0.02
*D′*, 𝜃	*S, S*′, *D*, ϕ	–0.46	0.01	–0.45	0.03
*D′*, ϕ	*S, S*′, *D*, 𝜃	–0.56	–0.01	–0.62	–0.01
*S′, S*	*D, D′*, 𝜃, ϕ	0.94	0.71	0.94	0.71
*D′, D*	*S, S′*, 𝜃, ϕ	0.56	0.08	0.62	0.09

*S, physical size; D, physical distance; S′, perceived size; D′, perceived distance; 𝜃, visual angle; ϕ, convergence angle*.

With respect to the two pairs with near zero partial correlations, that is, the *D* and *D*′, and ϕ and *D*′ pairs, it was ϕ for the *D* and *D*′ pair and *D* for the ϕ and *D*′ pair, respectively, that played mediating roles. Interestingly, the magnitudes of the two correlation coefficients were identical (0.56 for the *D* and *D*′ pair but -0.56 for the ϕ and *D*′ pair), reflecting the inverse relation between convergence angle and distance. The results of the correlation analyses indicate a strong relationship between *S* and *S*′, but relatively weaker relationships between the two distance pairs, i.e., *D*—*D*′ and ϕ—*D*′ pairs. In fact, all 18 participants reached the statistically significant level in the *S* and *S*′ pair with coefficients no lower than 0.81, whereas two participants failed to reach statistical significance levels in the *D*—*D*′ and ϕ—*D*′ pairs. The efficacy of convergence as a reliable source of distance information has been controversial with conflicting evidence (Heinemann et al., 1959; Foley, 1980; Collewijn and Erkelens, 1990; Brenner and van Damme, 1998). The current consensus is that convergence may, at best, be a rough indicator of distance at close range. Given that convergence angle was the only source of distance information available in the present setting, relatively poorer distance judgments may reflect this consensus view. It appears, nevertheless, that, except for a few individuals, most participants relied on the convergence angle to judge target distance in the present experiment.

Finally, with respect to the *S* and *S*′ pair, the strength of the relation remained high even with the effect of the candidate intervening variables factored out, clear evidence that *S*′ is determined exclusively by *S* alone. Taken together, these results are consistent with the thesis that the perceptions of size and of distance are two independent perceptual processes, and, by extension, that the binocular visual system perceives an object’s size directly by detecting information specified in a source of information, such as that proposed by Kim (2017).

Before pursuing the present issue further, the large errors observed in these two judgments must be investigated to determine whether they have any bearing on the main issue. The following facts may be relevant: first, errors were primarily constant and bias-induced. Second, participants had no practice trials prior to the experiment and received no feedback during the experiment. Finally, participants had to respond to virtual objects with which they had no prior experience. Thus it may be that the biased responses were the consequence of improper attunement or failure in calibration—or both—by the perception-action system (Bingham and Pagano, 1998; Withagen and Michaels, 2005; Jacobs and Michaels, 2006). Attunement refers to the detection of a specifying information source; whereas calibration refers to the scaling of perception to the information source detected (see Withagen and Michaels, for further details). Unfamiliarity with the task in conjunction with the lack of feedback during the experiment may have allowed the perception-action system to drift without a proper anchor. Still, it is worth recalling that the patterns of bias differed (overestimation of size but underestimation of distance). The following investigation was conducted to clarify whether the current results were due to lack of attunement or calibration on the part of the perception-action system. To this end, Experiment 2 replicated Experiment 1, except that a short practice session was provided prior to the experiment. Feedback was provided after each practice trial, but no feedback was provided during the experiment.

## Experiment 2: Practice Trials With Feedback

### Participants

Fifteen undergraduates (seven males and eight females) from Keimyung University volunteered for the experiment. None had participated in the previous experiment, and all were naïve to the purpose of the experiment. Participants received course credit for their participation. All participants had normal or corrected-to-normal vision, with stereoacuity of 100 s of arc or less.

### Apparatus

An NVIDIA 3D Vision^®^ toolkit was employed to present stereo images which were generated on a PC workstation equipped with an NVIDIA Quadro 2000 graphics card. The stereo images were displayed on a 22-in Samsung 2233RZ LCD monitor refreshed at 120 Hz. Participants viewed the displays in a dimly lit room through LC shutter glasses that were synchronized with the monitor’s refresh rate, which alternated at 60 Hz. The display had a resolution of 1680 H × 1050 V pixels and subtended a field of view of 43.3° H × 27.9° V when viewed from a distance of 60 cm. A head and chin rest was used to restrict head movements.

As in Experiment 1, the same black viewing box was placed between participants and the monitor and participants used the same apparatus to report responses.

### Procedure

The same procedure used in Experiment 1 was used in Experiment 2 except for a short practice session of nine trials that preceded the experiment. The nine trials were comprised of the following size and distance pairings (all units are in cm): (10.5, 41), (6.5, 46), (10.5, 36), (2.5, 46), (2.5, 41), (6.5, 41), (2.5, 36), (10.5, 46), and (6.5, 36). These pairs were presented in the same order to all participants. Participants responded by moving the distance block to the estimated distance and then making an aperture using their thumb and index finger, as in Experiment 1. After participants registered their distance judgments, the experimenter provided feedback by adjusting their hands to correspond to the actual distance. Similarly, after they registered their size judgments, the experimenter adjusted their finger aperture to correspond to the actual size of the object. During the feedback process, participants were instructed to maintain their gaze on the monitor and were not allowed to visualize the adjustments made. Therefore, the feedback was purely proprioceptive (i.e., tactile).

### Results and Discussion

**FIGURE 5:**
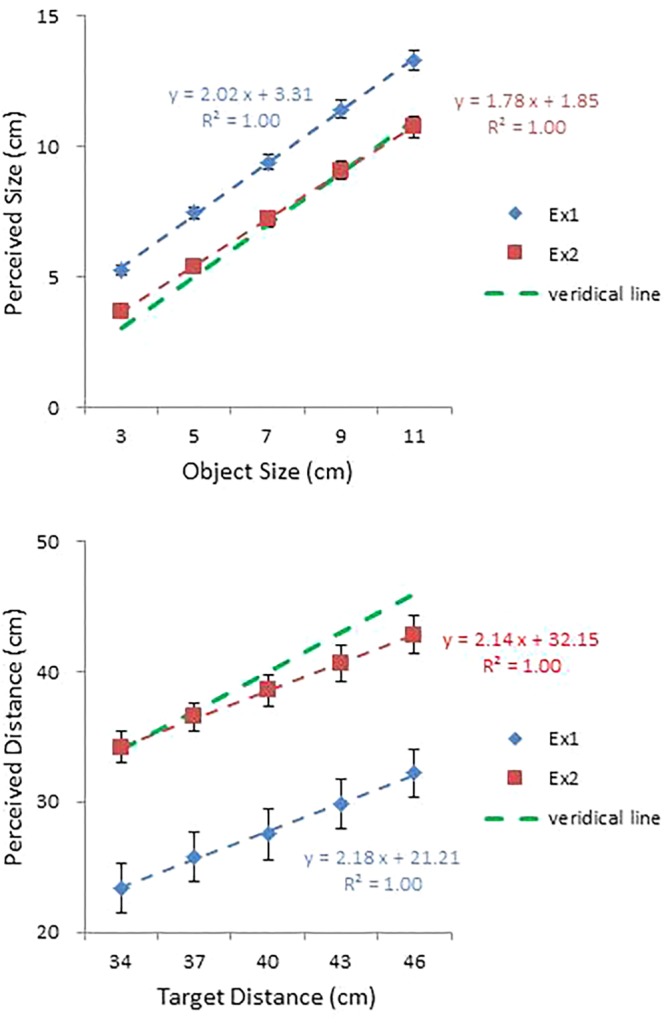
Mean perceived size plotted against object size **(top)** and mean perceived distance (with standard error bars) plotted against target distance **(bottom)** in Experiments 1 and 2. Regression lines are shown, together with the corresponding equations and *R*^2^ values. Error bars represent ±1 SE of the mean.

Mean perceived size and mean perceived distance are plotted against object size and target distance, respectively, in the top and bottom panels of **Figure 5**. For comparison, the corresponding data from Experiment 1 are also shown. Overall, accuracy improved substantially over the previous experiment. Mean constant errors in size and distance judgments were 0.20 and -1.33 cm, improvements of 0.20 and 11.23 cm, respectively. The effect of reattunement and/or recalibration is evident. Biases in size and distance judgments were reduced drastically, but more so with distance. It is remarkable that this change occurred after only nine practice trials with feedback. The near elimination of systematic errors confirms that performance in Experiment 1 was largely a result of miscalibration (i.e., inadequate scaling of the perceptual judgment) rather than failure in attunement (i.e., relying on non-specifying information source(s) for the judgment) (see Withagen and Michaels, 2005, for further discussion on the dissociation of attunement and calibration).

As in Experiment 1, a one-way repeated measures ANOVA on perceived size confirmed a significant effect of size, *F*(4,56) = 4.08, *p* < 0.01. A Tukey test further confirmed that all five sizes differed from each other. An ANOVA on perceived distance also confirmed a significant effect of distance, *F*(4,56) = 52.90, *p* < 0.0001, with all five distances differing from each other at the.05 level.

#### Constant Error Analysis

**FIGURE 6:**
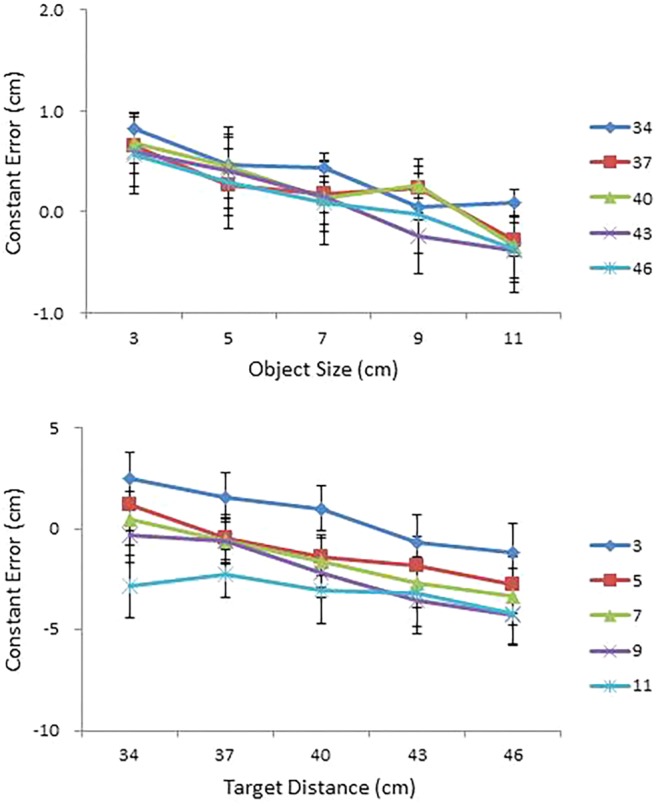
Mean constant error in perceived size as a function of object size (cm) for each condition of object distance (cm) **(top)**; and mean constant error in perceived distance as a function of object distance (cm) for each condition of object size (cm) **(bottom)** in Experiment 2. Error bars represent ±1 SE of the mean.

Mean constant error in perceived size and in perceived distance are presented, respectively, as a function of object size (cm) for each condition of object distance (cm) and as a function of object distance (cm) for each condition of object size (cm) in the top and bottom panels of **Figure 6**. As in Experiment 1, a repeated measures ANOVA was performed on constant error with size, distance, shape, and side as independent variables. The ANOVA on perceived size showed significant main effects of size, *F*(4,56) = 4.08, *p* < 0.01, $\eta_{p}^{2}$ = 0.23 and distance, *F*(4,56) = 3.16, *p* < 0.05, $\eta_{p}^{2}$ = 0.18. Object sizes were overestimated, but the degree of overestimation was greater at small sizes and at near distances. The ANOVA also confirmed a three-way interaction among size, shape, and side, *F*(4,56) = 2.75, *p* < 0.05, $\eta_{p}^{2}$ = 0.16.

An ANOVA on perceived distance showed main effects of size, *F*(4,56) = 12.94, *p* < 0.0001, $\eta_{p}^{2}$ = 0.48, and distance, *F*(4,56) = 8.69, *p* < 0.0001, $\eta_{p}^{2}$ = 0.38, which further interacted with each other, *F*(16,224) = 1.77, *p* < 0.05, $\eta_{p}^{2}$ = 0.11. Distance estimation decreased with increase in object size with overestimation of smaller objects and underestimation of larger objects. This pattern was particularly pronounced in the 3 and 7 cm size conditions, *F*(4,11) = 4.18, *p* < 0.05, and *F*(4,11) = 5.35, *p* < 0.05, respectively. The effect of object size on perceived distance was also pronounced in the 34, 40, and 46 cm distance conditions, *F*(4,11) = 5.37, *p* < 0.05, *F*(4,11) = 6.19, *p* < 0.01, and *F*(4,11) = 6.91, *p* < 0.01, respectively. The ANOVA also confirmed a significant four-way interaction among size, distance, shape, and side, *F*(16,224) = 2.10, *p* < 0.01, $\eta_{p}^{2}$ = 0.13.

In conjunction with the drastic reduction in the extent of errors in perceived size and perceived distance, response consistency also appears to have improved in Experiment 2, with the effects of shape and side less reliable in the present experiment and only observed in a three-way interaction in perceived size and a four-way interaction in perceived distance. The results of variable error analysis, which measures response consistency (Magill and Anderson, 2014), however, confirmed more consistency in perceived size in Experiment 2 (*M* = 0.14, *SD* = 0.03) than in Experiment 1 (*M* = 0.18, *SD* = 0.05), with the difference reaching statistical significance, *t*(31) = 2.53, *p* < 0.05, but not in perceived distance, *t*(31) = 1.71, *p* > 0.05.

#### Correlation Analysis

As in Experiment 1, the same bivariate and partial correlation analyses were performed for each participant; and the results are presented in **Table 1**. Visual inspection reveals that the results are nearly identical to those of Experiment 1. Bivariate correlations between 𝜃 and *S*′, *S* and *S*′, *D* and *D*′, and ϕ and *D*′ were strong but disappeared when the effect of confounding variables were controlled, except for the *S* and *S*′ pair. As in Experiment 1, it was *S* that confounded the strong bivariate correlation between 𝜃 and *S*′. With respect to the *D* and *D*′ and ϕ and *D*′ pairs, it was ϕ and *D*, respectively, that were the primary confounding variables for each pair, but to a lesser extent compared to Experiment 1. With respect to the *S* and *S*′ pair, by contrast, the strength of the correlation was retained even after the effect of control variables were removed, further confirming a direct causal relationship between the proposed information source for size and perceived size in binocular vision.

In summary, the results of Experiment 2 confirm that the errors observed in Experiment 1 were due to miscalibration arising from unfamiliarity with the task. Biases largely disappeared with nine practice trials accompanied by feedback. More importantly, the results of correlation analyses, taken together with nearly identical results of Experiment 1, strengthen the thesis that binocular perceptions of size and distance are two independent processes, not interdependent as the SDIH contends. Moreover, the results provide further evidence corroborating the utility of an information source such as that proposed by Kim (2017) for binocular judgments of size. More direct evidence was further pursued in Experiment 3.

## Experiment 3: Manipulating the Interpupillary Distance

The binocular source of information for object size proposed by Kim (2017) is comprised of two components, IPD and another composite term inside the square root sign. The composite term consists of four angular measures that are interlocked in a complex way. Thus, controlling any of these angular measures is not easy without perturbing the others. This leaves IPD as the only term that can be manipulated independently.

The effect of manipulating IPD on binocular perception is well known through studies using a telestereoscope, an instrument invented by Helmholtz in 1857 (Helmholtz, 1925; Finger and Wade, 2002). When viewed through a telestereoscope with an exaggerated IPD, the world appears shrunken (Judge and Bradford, 1988; Bennett et al., 1999, 2000). Conversely, with a diminished IPD, the world seems to enlarge. This is a widely practiced method to manipulate visual effects produced by stereoscopic display systems (Wartell et al., 1999). The exaggerated IPD enhances depth impressions, whereas the diminished IPD facilitates stereo fusion. The conventional explanation for these effects has been a change in convergence (or binocular disparity), which in turn scales object size in accordance with the SDIH. With the visual angle subtended by an object presumably left intact, convergence (or binocular disparity) would scale the visual angle to yield perceived distance consistent with the SDIH, thereby holding the perceived size of the object constant (i.e., size constancy), regardless of the telestereoscopic manipulation (Wallach et al., 1963; Kaufman, 1974).

**FIGURE 7:**
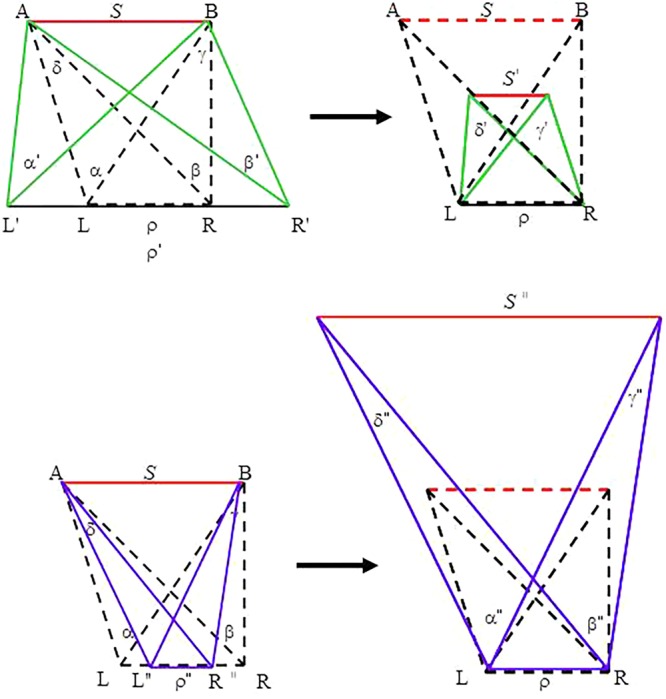
Geometries under altered IPDs: an exaggerated IPD (ρ′ > ρ) **(top)** and a diminished IPD (ρ″ < ρ) **(bottom)**. Dotted lines depict the geometries under the true IPD. The altered geometries (left panel) are scaled down or scaled up (right panel) to fit to the viewer’s true IPD. Thus, the exaggerated IPD must shrink (from ρ′ to ρ) **(top)**; whereas the diminished IPD must enlarge (from ρ″ to ρ) **(bottom)** to be viewed.

This explanation ignores the fact that heights appear exaggerated under an expanded IPD; whereas objects appear flattened (a phenomenon referred to as ‘cardboarding’ in 3-D photography) under a diminished IPD (Rule, 1941; Saxby, 2005). Moreover, if the user moves her head or an object moves, stereoscopic images shift and warp (Ware et al., 1998; see Wartell et al., 1999, for details; see also Wallach et al., 1963). Thus, changes in object size and in object distance are not the only effects of directly manipulating IPD on stereoscopic display systems. An alternative, but more systematic, account can be formulated based on the geometry employed to derive Kim’s (2017) binocular information source (**Figure 1B**). Consider a case of exaggerated IPD (from ρ to ρ′ with ρ < ρ′), as illustrated in the top panel of **Figure 7**. Here, the four angular measures change concurrently with the change in the IPD. To be viewed by the viewer with IPD = ρ, the whole geometry must scale down (i.e., shrink) to fit to the viewer’s IPD. For a diminished IPD (from ρ to ρ″ with ρ > ρ″), as shown in the bottom panel of **Figure 7**, the reconfigured geometry must scale up (i.e., enlarge) in accordance to the viewer’s IPD. In brief, telestereoscopic manipulation yields perceptual effects that alter, not only the perceived size of an object (object size *S* decreases to *S*′ in the case of exaggeration but increases to *S*″ in the case of contraction) and its perceived distance from the viewer, but also the entire geometry—including all the angular measures. This geometric reconfiguration may cause the distorted perception reported in the literature.

Note that the previous research utilizing a telestereoscope was for its enhanced depth impression and subsequent adaptation under the modified viewing condition (Wallach and Karsh, 1963; Wallach et al., 1963; Epstein, 1968; Bennett et al., 1999, 2000). The present experiment, by contrast, explored the effects of the modified IPD, not only on distance judgments, but also on size judgments both under exaggerated and contracted conditions. With the proposed information source by Kim (2017), it is possible to make specific predictions about the effects of the modified IPD on size and distance judgments, making this study unique. Thus, these effects will be utilized to assess the utility of the proposed information source. Note that of the many studies that examined the effects of the telestereoscope on perception, no quantitative or qualitative predictions were ever offered *a priori*. The effects were simply noted in *post hoc* analyses.

### Participants

Fifteen experimentally naïve participants, all graduates (six males and nine females) from Keimyung University, volunteered for the experiment. All participants had normal or corrected-to-normal vision, with stereoacuity of 100 s of arc or less.

### Stimuli

The apparatus and viewing geometry used in Experiment 1 were again used in Experiment 3. However, objects appeared only as cubes.

### Design

Four variables were manipulated. The simulated size of the cube varied among 3.6, 5.5, 7.4, and 9.3 cm; and its simulated location varied among 35, 37, 39, and 41 cm from the observation point. The IPD values varied among 0.8, 1.0, and 1.2. Each participant’s true IPD value (1.0) was either diminished by 20% (IPD = 0.8) or exaggerated by 20% (IPD = 1.2). In accordance with the proposed information source, it was expected that perceived size and distance would increase by 25% (1/0.8 = 1.25) at IPD = 0.8; but decrease by 17% (1/1.2 = 0.83) at IPD = 1.2. As in the previous experiments, each cube was centered 3–4 cm to the left or to the right of the center of the screen. These manipulations yielded a 4 (Size) × 4 (Distance) × 3 (IPD) × 2 (Side) repeated measures design with 96 completely randomized trials.

### Procedure

As in Experiment 2, a short practice session preceded the main experiment to minimize the biased responses observed in Experiment 1. The practice trials were comprised of the following ten pairs of size and distance (all units are in cm), all rendered using each participant’ true IPD value (IPD = 1.0): (9.6, 47), (6.4, 45), (4.0, 33), (7.2, 49), (8.0, 31), (3.2, 37), (10.4, 41), (5.6, 39), (4.8, 35), and (8.8, 43). Practice trials were followed by feedback, as in Experiment 2, and were presented in the same order to all participants. No feedback was provided during the experiment.

### Results and Discussion

**FIGURE 8:**
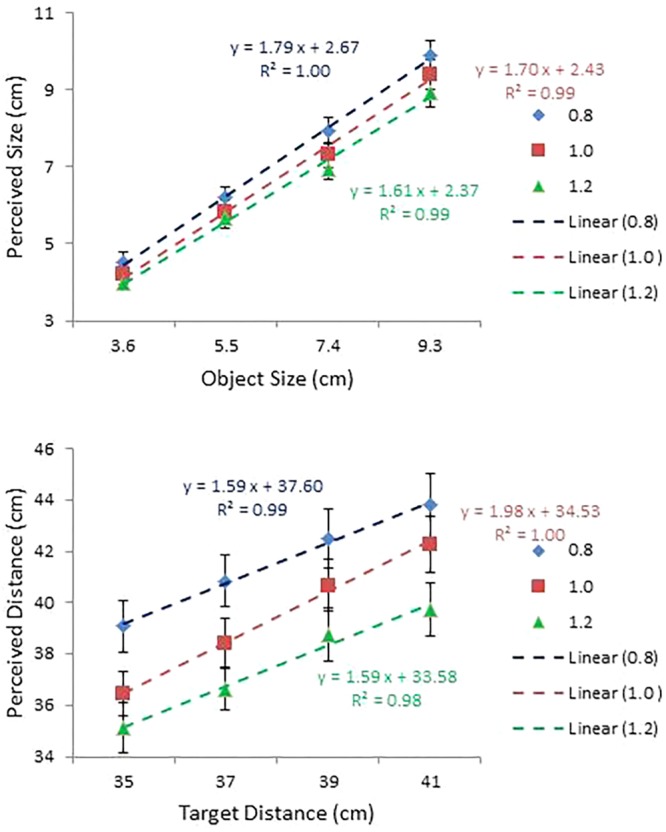
Mean perceived size plotted against object size **(top)**; and mean perceived distance plotted against object distance **(bottom)** for each condition of IPD used in Experiment 3. Regression lines are shown, together with the corresponding equations and *R*^2^ values.

Perceived size and distance are plotted against object size and object distance, respectively, for each condition of IPD in the top and bottom panels of **Figure 8**. Judgments under the two altered IPD conditions were ordered as predicted, but not to the extent predicted. Perceived size and distance were expected to increase by 25% at IPD = 0.8; but they increased, on average, by 13 and 9%, respectively. On the other hand, they were expected to decrease by 17% at IPD = 1.2, but instead perceived size increased by 1% and perceived distance decreased by 1%. To cite specific examples, for a 7.4 cm size object under the 0.8 and 1.2 IPD conditions participants were expected to report sizes of 9.25 (i.e., 7.4/0.8) and 6.17 (i.e., 7.4/1.2) cm, respectively. Instead, the average judged values were 7.93 and 6.95 cm. For the 39 cm condition, under the two altered IPD conditions expected values were 48.75 and 32.50 cm, respectively, but the average judged values were 42.52 and 38.74 cm. In short, the effect of scaling was less pronounced than expected. Probably the weak effect of IPD manipulation has to do with the fact that the effect was virtual produced by graphic simulation rather than actual experienced watching through real telestereoscopes. Nevertheless, judgments were ordered as predicted. That is, compared with judgments at IPD = 1.0, judgments at IPD = 0.8 were bigger (perceived size) and farther (perceived distance), while judgments at IPD = 1.2 were smaller and nearer.

These results are not unusual, however. Judge and Bradford (1988) examined one-handed ball catching performance under telestereoscopic viewing. The telestereoscope used in their study increased IPD by 8.0 cm. With this manipulation, they expected that the perceived position of the ball would shrink by a factor of 2.3 for a typical individual with an IPD of 6.2 cm (14.2/6.2 = 2.3). The effect of telestereoscopic viewing was dramatic. All their participants closed their hands too early, suggesting that perceived distance shrank substantially. Nevertheless, perceived distance did not correspond precisely to the predicted scale factor. When the perceived ball position was converted into a scale factor, the observed scale factor was less than the predicted one, a pattern comparable to that observed in the present experiment (see also Wallach and Karsh, 1963, for a similar finding).

Overall, participants responded in a manner consistent with expectation. This observation was confirmed by two separate ANOVAs. The ANOVA on perceived size with IPD and object size as within-subject factors confirmed main effects of IPD, *F*(2, 28) = 65.61, *p* < 0.0001, $\eta_{p}^{2}$ = 0.82, and size, *F*(3,42) = 323.97, *p* < 0.0001, $\eta_{p}^{2}$ = 0.96. All means for perceived size at the 3 IPD values differed from each other at the 0.001 level, as did the means for the four object sizes. The ANOVA also confirmed a significant interaction between IPD and size, *F*(6,84) = 3.75, *p* < 0.01, $\eta_{p}^{2}$ = 0.21. However, a simple effects analysis showed IPD was significant at all four sizes, as was size at all three IPDs. Although the result of this analysis was non-specific about the source of this interaction, a comparison of the slopes of the regression lines (slope = 0.94 at IPD = 0.8; 0.90 at 1.0; and 0.85 at 1.2; see the top panel of **Figure 8**) reveals that the effect of the IPD manipulation on perceived size was progressively more pronounced as object size increased.

The ANOVA on perceived distance, with IPD and distance as within-subject factors, revealed main effects of IPD, *F*(2,28) = 45.86, *p* < 0.0001, $\eta_{p}^{2}$ = 0.77, and distance, *F*(2,28) = 62.23, *p* < 0.0001, $\eta_{p}^{2}$ = 0.82. The interaction between IPD and distance was not significant, *F* < 1, *ns*. As for perceived size, all means of perceived distance at the 3 IPD values differed from each other at the 0.001 level, as did the means at the four distances.

#### Constant Error Analysis

**FIGURE 9:**
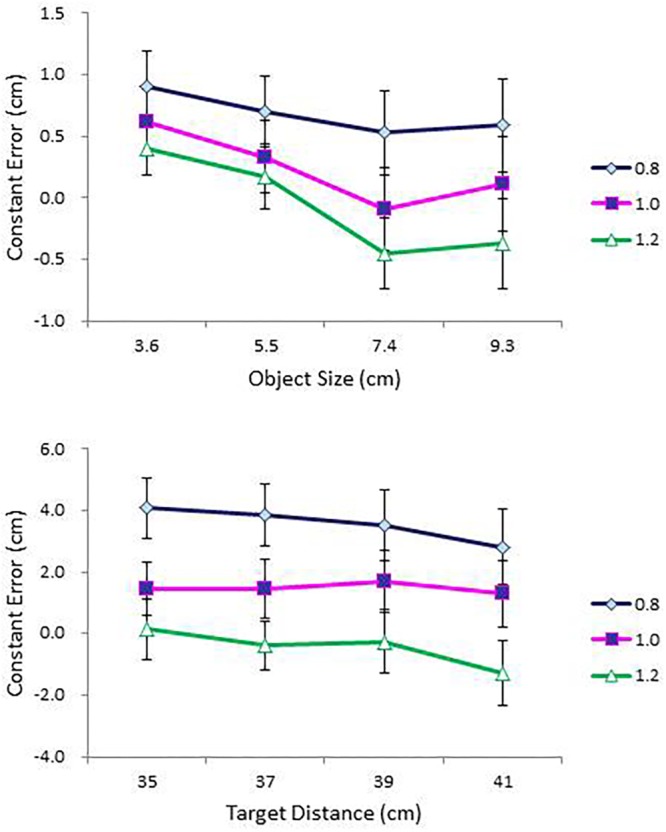
Mean constant error in perceived size as a function of object size (cm) **(top)**; and mean constant error in perceived distance as a function of object distance (cm) **(bottom)** for each condition of IPD in Experiment 1. Error bars represent ±1 SE of the mean.

As in the previous experiments, judgment accuracy was assessed using constant error. Mean constant error in perceived size and in perceived distance are presented as a function of object size (cm) and as a function of object distance (cm), respectively, for each condition of IPD in the top and bottom panels of **Figure 9**. An ANOVA on perceived size with size, distance, IPD, and side as within-subject factors showed main effects of size, *F*(3,42) = 5.75, *p* < 0.01, $\eta_{p}^{2}$ = 0.29, and distance, *F*(3,42) = 8.04, *p* < 0.0001, $\eta_{p}^{2}$ = 0.37. Overestimation at 3.6 cm (*M* = 0.64) differed significantly from slight underestimation at 7.4 cm (*M* = -0.01) and slight overestimation at 9.3 cm (*M* = 0.11). In addition, overestimates of size were greater in the nearest (i.e., 35 cm) condition (*M* = 0.49) than in the two distant (i.e., 39 and 41 cm) conditions (*M* = 0.18 and *M* = 0.16, respectively).

Importantly, the ANOVA confirmed a main effect of IPD, *F*(2,28) = 65.46, *p* < 0.0001, $\eta_{p}^{2}$ = 0.82, and its interaction with size, *F*(6,84) = 3.75, *p* < 0.01, $\eta_{p}^{2}$ = 0.21 (top panel of **Figure 9**). As expected, object size was severely overestimated at IPD = 0.8 (*M* = 0.68), less so at IPD = 1.0 (*M* = 0.24), and slightly underestimated at IPD = 1.2 (*M* = -0.06). A simple effects analysis of the IPD × Size interaction confirmed this result. The effect of IPD was significant in each size condition. By contrast, the effect of size differed across the three values of IPD. In particular, its effect did not reach significance at IPD = 0.8, but reached significance at IPD = 1.0, *F*(3,12) = 13.97, *p* < 0.0001, and at IPD = 1.2, *F*(3,12) = 11.48, *p* < 0.01. Object size was overestimated at two small sizes (i.e., 3.6 and 5.5 cm) and either underestimated or slightly overestimated at two large sizes (i.e., 7.4 and 9.3 cm). Overestimation of size, even at IPD = 1.0 (i.e., participants’ true IPD values), is consistent with Experiment 2, in which object sizes were overestimated across all conditions of size, even after bias correction.

The ANOVA on perceived distance showed main effects of size, *F*(3,42) = 13.31, *p* < 0.0001, $\eta_{p}^{2}$ = 0.49, IPD, *F*(2,28) = 45.86, *p* < 0.0001, $\eta_{p}^{2}$ = 0.77, and side, *F*(1,14) = 4.66, *p* < 0.05, $\eta_{p}^{2}$ = 0.25. Although distances were overestimated across all size conditions, overestimation in the two small size conditions was particularly pronounced. Judgments in the 3.6 cm condition differed significantly from those in the other three size conditions; and judgments in the 5.5 cm condition differed from those in the 9.3 cm condition. Again, consistent with the expectation, distances were severely overestimated at IPD = 0.8 (*M* = 3.56), less so at IPD = 1.0 (*M* = 1.47) and underestimated at IPD = 1.2 (*M* = -0.44), with all three means differing from one another at the 0.001 level. The effect of side replicated the same effect observed in Experiment 1. Objects on the left-hand side were more overestimated (*M* = 1.83) than those on the right-hand side (*M* = 1.23).

#### Correlation Analysis

As in the previous experiments, the same bivariate and partial correlation analyses were performed for each participant; and the results are presented in **Table 2**. Remarkably, the results nearly replicated those of the two previous experiments with high bivariate correlations between 𝜃 and *S*′, *S* and *S*′, *D* and *D*′, and ϕ and *D*′, which all disappeared when the effects of control variables were removed under partial correlations except for the *S* and *S*′ pair. As in the previous experiments, the strong bivariate correlation between 𝜃 and *S*′ turned out to be spurious, arising primarily from high correlation of each member of the pair with *S*. Again, as in the previous experiments, the strong relationships between the *D* and *D*′ and ϕ and *D*′ pairs resulted from the confounding effects of ϕ and *D*, respectively, but to lesser extents compared to Experiments 1 and 2. Weak distance signals conveyed by convergence angle appear to be further compromised under IPD manipulation. By contrast, the strength of the *S* and *S*′ pair remained high—even after the removal of the effects of control variables, replicating the preceding results. This finding is particularly significant, considering that the researchers who employed a telestereoscope to examine its effect on binocular perception attributed distorted perception to a change in convergence by virtue of the increased IPD (Wallach et al., 1963; Kaufman, 1974; Judge and Bradford, 1988; Bennett et al., 1999, 2000). Likewise, the distorted distance judgments observed in the present experiment can be attributed to the change in convergence introduced by manipulating IPD. Visual angle, however, was unaffected even under the IPD manipulation. If perceptions of size and distance are interdependent as the SDIH contends, altered distance judgments must have impacted size judgments equally. No such evidence was observed. On the contrary, perceived size not only remained strongly coupled to *S*, but also altered in proportion to the extent of change in IPD. Note that the four angles constituting Kim’s (2017) binocular variable reconfigures automatically in compliance with the change in IPD, and requisite information about altered size is always made available to the binocular visual system for accurate judgments. This result, therefore, can only be accounted for by Kim’s (2017) binocular variable.

**Table 2:** Mean bivariate and partial correlation coefficients among stimulus and perceptual variables under each condition of IPD in Experiment 3.

		ipd = 0.8		ipd = 1.0		ipd = 1.2	
Paired variables	Controlled variables	Bivariate correlation	Partial correlation	Bivariate correlation	Partial correlation	Bivariate correlation	Partial correlation
*S′, D′*	*S, D*, 𝜃, ϕ	–0.23	–0.12	–0.31	–0.02	–0.32	–0.07
*S′*, 𝜃	*S, D, D′*, ϕ	0.84	0.14	0.85	0.13	0.83	0.09
*S′*, ϕ	*S, D, D′*, 𝜃	0.06	0.06	0.07	0.11	0.07	0.03
*D′*, 𝜃	*S, S*′, *D*, ϕ	–0.30	0.09	–0.38	0.16	–0.35	0.13
*D′*, ϕ	*S, S*′, *D*, 𝜃	–0.44	–0.05	–0.50	–0.02	–0.39	–0.05
*S′, S*	*D, D′*, 𝜃, ϕ	0.91	0.64	0.92	0.64	0.91	0.60
*D′, D*	*S, S′*, 𝜃, ϕ	0.45	–0.02	0.50	0.06	0.39	–0.02

*S, physical size; D, physical distance; S′, perceived size; D′, perceived distance; 𝜃, visual angle; ϕ, convergence angle*.

Reinforcing the present conclusion is the fact that the effect of telestereoscopic manipulation is largely on the horizontal dimension, with minimal impact on the vertical dimension. As noted above, Judge and Bradford (1988) reported the perceived ball position along the depth axis shrank under telestereoscopic viewing although the extent of distortion did not conform to the predicted scale factor. Interestingly, however, distortion along the vertical axis did not differ from the ‘normal’ value with large standard errors (see also Wallach et al., 1963, for a similar finding). This finding is consistent with the observation that, when viewing a telestereoscope with an enlarged IPD, the heights of objects appear exaggerated (Saxby, 2005). The four angles comprising Kim’s (2017) proposed variable are horizontal. Any modification of the IPD would perturb all four angular measures concurrently (see **Figure 7**), thereby distorting the overall computation. The pattern of distortion (i.e., distortion confined along the depth axis but intact along the vertical axis) reported by Judge and Bradford is consistent with the proposed binocular variable.

To summarize, the effect of manipulating IPD was dramatic. Under the exaggerated IPD (IPD = 1.2), objects were perceived smaller and closer than they were with the true IPD (IPD = 1.0). The pattern reversed, however, with the diminished IPD (IPD = 0.8), as objects were perceived larger and farther away than with the true IPD. Yet, the results conformed to the predictions derived from Kim’s (2017) proposed variable. These results, taken together with the results of correlation analyses, demonstrate the perceptual independence of size and distance and, at the same time, provide strong support for the utility of the binocular information source proposed by Kim (2017).

## General Discussion

The present study was motivated by possible differences between monocular and binocular modes of visual perception and their implications for size perception. Specifically, this study sought to clarify whether the visual system relies on a generic solution such as the SDIH for a visual task, i.e., size perception, irrespective of the mode of perception, or utilizes a mechanism designed specifically for the specific mode of perception such as that proposed by Kim (2017) for binocular size perception.

Three experiments were carried out to address this issue. Kim’s proposed variable is comprised of four angular measures and the IPD with explicit exclusion of egocentric distance information. This variable, therefore, necessitates independence of perceived size and perceived distance, whereas the SDIH predicates interdependence of the two percepts. The first two experiments examined whether perceived size and perceived distance are independent, as entailed by Kim’s binocular variable, or interdependent, as the SDIH assumes. Participants viewed a virtual object stereoscopically and judged its size and distance. Participants’ size judgments were more accurate and less biased than their distance judgments. The results of partial correlation analyses were more straightforward with no evidence implicating the SDIH for size perception under binocular viewing. Instead, the results were unequivocal, demonstrating that the perceptions of size and of distance are two independent perceptual processes with each determined directly by the corresponding information source. Distance judgments were comparatively poorer, possibly reflecting weak distance signals conveyed by convergence. By contrast, perceived size and (manipulated) object size maintained a strong correlation even after the effect of other potential distance and size cues were factored out, a clear demonstration that object size was perceived directly by a variable conveying metric size information, i.e., Kim’s binocular variable. To provide further evidence for the proposed information source, Experiment 3 explicitly manipulated participants’ IPDs, one of its components. Consistent with the prediction derived from the proposed variable, size and distance judgments were overestimated under a diminished IPD but underestimated under an enlarged IPD. More importantly, the patterns of correlations were nearly identical to those observed in the previous experiments, further reinforcing the conclusion that each percept is directly linked to the corresponding information source.

As underscored in Kim (2017), the proposed information source is unique because it is one of the few sources of information identified to date that is capable of conveying absolute metric information about a spatial dimension for binocular vision. Another feature of the information source that attracts attention is that it combines two measures of binocular parallax with two measures of visual angle and the IPD. The inclusion of binocular parallax (in essence, the angle of convergence) in the variable is notable considering that the model is proposed as a source of information for size perception. Indeed, the assumption that the convergence angle is a source of distance information goes as far back as Descartes and Bishop Berkeley. The proposed variable suggests that the convergence angle can be better construed as a facilitator of size perception. Gillam (1995) recognized the conflicting evidence arising from research directed at determining whether size is a derived quantity based on distance information as contended by the SDIH or should be recognized as a primary perceptual quality such as motion. The latter conception remained as a conjecture, primarily due to lack of a suitable information source for size perception. Gillam further speculates that oculomotor signals may have greater facilitating effects on size perception than on distance perception. Kim’s proposed variable provides strong support for Gillam’s conjecture that oculomotor signals may contribute more to size perception than to distance perception.

In conclusion, size perception is ubiquitous in our lives; and we make the necessary perceptual judgments with precision and seemingly little effort. Attempting to account for this remarkable perceptual capacity has a history dating back to Euclid, Ptolemy, Alhazen, Descartes, Berkeley, Helmholtz, and many more (Ross and Plug, 1998; Hatfield, 2002). The prevalent explanation for this phenomenon has been the SDIH. Mounting evidence, however, has questioned the SDIH’s validity (Heinemann et al., 1959; Oyama, 1974; Foley, 1980; Sedgwick, 1986; Collewijn and Erkelens, 1990; Brenner and van Damme, 1998; Haber and Levin, 2001; Kim et al., 2016, to name a few). Yet, the SDIH has endured. Lack of identified information sources for size perception may have contributed to its enduring popularity (Kim, 2017). The present results, however, provide convincing evidence eliminating the SDIH as an explanation for size perception under binocular viewing, adding another piece of evidence to the vast literature that demonstrated independence of perceived size and distance. The question remains as to whether the SDIH still has utility for monocular vision. Thus, it is incumbent upon proponents of the SDIH to assess its utility using an experimental paradigm similar to that employed in the present study. Significantly, the present results provide strong evidence corroborating the utility of the information source proposed by Kim (2017). Nevertheless, the finding that judgments under the altered IPD conditions in Experiment 3 were ordered as predicted, but not in terms of absolute magnitude, caution its overgeneralization. Clearly, more research is needed to further confirm the utility of the proposed information source for the binocular visual system.

## Author Contributions

The author confirms being the sole contributor of this work and approved it for publication.

## Conflict of Interest Statement

The author declares that the research was conducted in the absence of any commercial or financial relationships that could be construed as a potential conflict of interest.

## Footnotes

1 It is worth noting that all participants in this experiment were capable of fusing the stereo images with little difficulty, as shown by their observed accuracy in the experimental task. As noted above, all of the virtual images were rendered in crossed disparity; hence, appeared as if floating in front of the monitor. Accordingly, consistent with the finding first reported by Wheatstone (1852), all images rendered on the monitor were larger than the virtual images perceived by the participants. For example, a 3 cm (5.05°) object shown at 34 cm from the observer rendered on the monitor as two 5.3 cm images. Had the participants not fused the images properly, their performance would have been severely affected by the actual images on the monitor.

2 The condition that induces these images is occlusion; and its effect on binocular vision is described variously as gain-or-loss disparity (Barrand, 1979) or da Vinci stereopsis (Nakayama and Shimojo, 1990; Cook and Gillam, 2004).

3 Constant error is the signed deviation from the target, thus measures the directional bias of response errors (Magill and Anderson, 2014).

4 For monocular vision, the visual angle subtended by a viewed object is defined with respect to each eye. For binocular vision, the visual angle subtended at each eye would be identical had the viewed object been positioned at the center of the two eyes. The viewed object in the present study (the cube) was shifted laterally from the center by a random amount to minimize the familiarity effects, thereby rendering the two visual angles different. For this reason, the visual angle was defined, for the sake of computation, with respect to the midpoint of the IPD.

5 It is the case that, when measured from the origin of the distance ruler, the objects on the left side are a little farther away than those on the right side. It is not clear, however, whether the effect of side reflects this geometric arrangement.
